# Occupational health legislation and practices related to seafarers on passenger ships focused on communicable diseases: results from a European cross-sectional study (EU SHIPSAN PROJECT)

**DOI:** 10.1186/1745-6673-5-1

**Published:** 2010-02-10

**Authors:** George Rachiotis, Varvara A Mouchtouri, Clara Schlaich, Tobias Riemer, Carmen Varela Martinez, Gordon Nichols, Christopher LR Bartlett, Jenny Kremastinou, Christos Hadjichristodoulou

**Affiliations:** 1 Department of Hygiene and Epidemiology, Faculty of Medicine, University of Thessaly, 22 Papakiriazi Str., Larissa, 41222, Greece; 2 Hamburg Port Health Center, Institute of Occupational and Maritime Medicine, Seewartenstrasse 10 20459 Hamburg, Germany; 3 National Centre of Epidemiology, Sinesio Delgado 6 28029, Madrid, Spain; 4 Gastrointestinal, Emerging and Zoonotic Infections Department Health Centre for Infections, Health Protection Agency, 61 Colindale Avenue, London NW9 5EQ, UK; 5 UCL Centre for Infectious Disease Epidemiology Department of Primary Care Population Sciences Royal Free and University College Medical, School Latchmoo Farm, Tile Barn Lane, Brockenhurst, SO42 7UE, UK; 6 Department of Public and Administrative Health, National School of Public Health, 196 Leoforos Alexandras, 115 21 Athens, Greece

## Abstract

**Background:**

Seafarers play an important role in the transmission of communicable diseases. The aim of the present study is to draw information and identify possible gaps on occupational health practices related to seafarers sailing on ships within the European Union Member States (EU MS) with focus on communicable diseases.

**Methods:**

A structured questionnaire was sent to competent authorities from 21 EU MS. The questionnaire included questions about occupational health policies, medical certification of seafarers, communicable diseases reporting and relevant legislation. Descriptive analysis of the data was conducted by the use of Epi Info software: EU MS were categorized in four priority groups (A, B, C, D) based on: number of passenger ships visits, volume of passengers, and number of ports in each country. Moreover, EU MS were categorized to old and new, based on the date of entry in the EU.

**Results:**

All 21 countries with relevant competent authorities responded to the questionnaire. The existence of specific national legislation/regulation/guidelines related to vaccination of seafarers was reported by three out of the 21 (14%) responding authorities. Surveillance data of communicable diseases related to seafarers are collected and analyzed by 4 (19%) authorities. Five out of 21 of the responding countries (24%) reported that tuberculin test result is required for the issuance of seafarer's medical certificate while a great variety of medical examination is required for the issuance of this certificate among countries.

Gaps on occupational health services focused on communicable diseases related to maritime occupation have been reported by 33% of the responding countries.

Responding authorities from Group A and B had the highest percentage of reported gaps followed by groups C and D. Old MS reported a higher frequency regarding gaps on occupational health services in comparison to new MS.

**Conclusion:**

Our results revealed heterogeneity regarding occupational health of maritime employees in EU MS. This work provides some evidence that further work at international and European level could be considered, in order to explore the potential for harmonized initiatives regarding occupational health of seafarers.

## Background

The shipping industry has grown rapidly in recent years, and dramatic increases in size and passenger capacity have been recorded. The cargo shipping industry is also growing. The world merchant fleet comprises 1.4 million seafarers of whom two thirds work within multiethnic crews. It is expected that twenty million people will sail on cruise ships in the year 2010 [1]. Moreover, the morbidity of maritime employees in the period of globalization is an important issue for occupational health care in the shipping industry [2,3].

It is widely accepted that seafaring is considered a high risk job in terms of health and safety at work, while provision of health care aboard is a very complex question [4]. Seafarers by nature of their work are exposed to a variety of occupational hazards making exposure to biological agents and the concomitant risk of communicable diseases extremely important within this working group [5]. Maritime employees can travel to various geographical areas, far away from their own countries. Consequently, they are at risk of contracting infectious diseases at ports of call in different countries. In 1993 the common committee of International Labor Organization (ILO), and World Health Organization (WHO) identified Hepatitis B virus infection (HBV), Immunodeficiency Virus infection (HIV), and Acquired Immunodeficiency Syndrome (AIDS) as infectious diseases against which there should be provisions for guidance on prevention [6]. Furthermore, the possibility that a seafarer may transmit biological agents to other persons, could be associated with public health implications, and contribute to trans-national transmission of communicable diseases.

The framework of pre-medical examination of seafarers is given by international conventions, national statutory systems and company requirements. The requirement for seafarers to have a certificate of medical fitness is stipulated in general terms on ILO and International Maritime Organisation (IMO) conventions - most recently in the Maritime Labour Convention of 2006 [7,8]. The WHO collaborated with ILO to produce the 1997 Guidelines for Conducting Pre-sea and Periodic Medical Fitness examinations [9]. These international conventions are addressed to the maritime regulatory bodies in the countries that ratify the conventions. Each country then produces its own regulations that have to meet the minimum standards in the conventions but may add additional requirements.

There is currently no legal or technical framework produced by the European Council in the criteria for the fitness assessment of seafarers.

Epidemiological research on maritime employees has been concerned - mainly-with national studies. However, the need for international studies has been pointed out [10]. Up to now there is no published information on occupational health legislation and practices related to maritime employees with emphasis on communicable diseases which is applied by the competent authorities of EU MS.

The aim of our work within the context of the SHIPSAN project was to draw information from competent authorities of EU MS regarding legislation and practice related to seafarer's occupational health and gaps on occupational health services provided to seafarers focused on communicable diseases.

## Methods

The survey was performed within the EU SHIPSAN project. A preliminary questionnaire was sent to all EU MS in order to identify the relevant competent authorities. Thereafter, a detailed questionnaire was constructed and sent to competent authorities in 21 countries (which were identified from the preliminary questionnaire). The questionnaire included questions on: a) national legislation or regulation, or guideline for occupational health of seafarers, b) the existence of additional institutions next to the national public health institutions where communicable diseases of seafarers are reported, c) the collection and central analysis of surveillance of communicable diseases data related to seafarers, d) the vaccination of maritime employees, beside Yellow fever which is mandatory under IHR 2005 [11], e) the issuance of seafarer's certificates, f) medical examination of food handlers who are employed to work on ships and gaps on occupational health services provided to seafarers with focus on communicable diseases (additional file 1).

EU MS were categorized in four priority groups (A, B, C, D) based on: number of passenger ships, volume of passengers, and number of ports. Moreover, EU MS were categorized to old and new based on the date of entry in the European Union. The questionnaire was pilot tested in Germany and Malta. In order to ensure data collection from the high priority group A of EU MS, site visits were organized. Data collected were entered in an electronic database. Descriptive analysis was conducted, and Fischer's exacts test was used as the Univariate analysis for comparisons between different groups. Statistical analysis was performed by the use of Epi-Info software. The level of statistical significance was <0.05.

## Results

Five countries didn't respond to the preliminary questionnaire, and three countries reported no competent authority.

Twenty one MS reported the existence of a national competent authority and all participated in the study.

Four out of 21 (19%) EU MS (Croatia, Malta, Italy and Poland) reported the existence of additional institutions next to the national public health institutions where communicable diseases of seafarers are reported to Table 1. These countries more often reported the existence of specific national legislation or regulation or guidelines related to vaccination of seafarers in comparison to countries without these additional institutions.

**Table 1:** Occupational health legislation and practices in the EU MS.

Subject area			Yes/Total	Percent
Specific National Legislation or regulation or guideline for Occupational Health of seafarers except for EU or International Maritime Organisation or of the International Labour Organisation?			11/21	52.4%

Additional Institutions next to the national public health institutions where Communicable diseases of seafarers are reported			4/21	19%

Collection and analysis of surveillance of communicable disease data related to seafarers			3/21	14.2%

Specific national legislation or regulation or guideline for vaccination of Seaferers			3/21	14.2%

Additional National Recommendations for vaccinations of seafarers except those required by the IHR?			5/21	24%

	Are Medical certificates obligatory in order for seafarers to travel?		20/21	95.2%
	
Medical Certificates:	Medical Examinations Required	History of communicable diseases plus clinical examinations	17/21	81%
		
		Vision function tests	20/21	95.2%
		
		Hearing function tests	20/21	95.2%
		
		Dental Check	13/21	61.9%
		
		Blood Tests	12/21	57.1%
		
		X-Rays	14/21	66.7%
		
		Tuberculin tests	5/21	23.8%
		
		Other	11/21	52.4%

Do the medical certificates have time frame duration?			21/21	100%

In your opinion are there any gaps in occupational health services focused in communicable diseases related to maritime occupation			7/21	33.3%

Three out of 13 (23%) of the responders of Groups A and B reported that the surveillance data of communicable diseases related to seafarers are collected and centrally analyzed but none in the groups C and D (Table 2). Eleven out of 21 (52.4%) of the responding countries reported the existence of specific national legislation or regulation or guideline for seafarer's occupational health beside the EU or International Maritime Organization or of the International Labor Organization directives or conventions. Analysis of the results by priority group showed that 8 out of 13 (61.5%) EU MS responding authorities (belonging to Groups A and B) reported the existence of specific national legislation related to seafarer's occupational health, while only 37.5% from Groups C and D reported the existence of such legislation (Table 2).

**Table 2:** Analysis of the results by priority group

	Priority groups				
		
Question	(A+B)		(C+D)		p value
		
	Yes/Total	%	Yes/Total	%	
Is there any specific national legislation or regulation or guidelines related to occupational health of seafarers except for that of EU or of the International Maritime Organisation (IMO) or of the International Labour Organization (ILO)?	8/13	61.5%	3/8	37.5	0.26

Are the surveillance of communicable diseases data related to seafarers collected and centrally analysed?	3/13	23%	0/8	0	0.25

Is there any specific national legislation or regulations or guidelines related to vaccination of seafarers?	3/13	23%	0/8	0	0.25

Dental check	10/13	76.9%	3/8	37.5	0.08

Blood tests	6/13	46.2%	6/8	75	0.20

Tuberculin tests	4/13	30.8%	1/8	12.5	0.34

In your opinion are there any gaps in occupational health services focused in communicable diseases related to maritime occupation?	6/13	46.2%	1/8	12.5	0.17

Overall, only five out of 21 responders (24%) reported the existence of additional national recommendation for vaccination of seafarers except for Yellow Fever which is mandatory under the International Health Regulation 2005. New EU MS reported the existence of additional institutions next to the national public health institutions where communicable diseases related to maritime occupation are reported more often compared to old EU MS (30%; versus 9.1% respectively; Table 3). Only three out of 21 (14.2%) of the EU MS which participated in the survey stated that national data of surveillance of communicable diseases related to seafarers were collected and centrally analyzed.

**Table 3:** Comparison of the results between Old and New Member States *

	Member States				
		
Question	New		Old		P value
		
	Yes/Total	%	Yes/Total	%	
Are there additional institutions next to the national public health institutions where communicable diseases of seafarers are reported to?	3/10	30	1/11	9.1	0.31

Blood tests	9/10	90	3/11	27.3	0.005

Tuberculin tests	1/10	10	4/11	36.4	0.18

In your opinion are there any gaps in occupational health services focused in communicable diseases related to maritime occupation?	2/10	20	5/11	45.5	0.36

Regarding seafarers medical certificate, required medical examinations and tests vary between nations: Vision and hearing function tests are performed by 84,6% of the responders while X-Rays, blood tests, and tuberculosis skin test were performed by 57.7%, 50.0%, and 19.2%, respectively. Thirteen (61.9%) countries required dental check of seafarers (Table 1). Four out of 13 (30.8%) of the countries from priority groups (A and B) reported that tuberculin test result were required for the issuance of seafarer's medical certificate, while only one out of 8 (12.5%) of the countries from Groups C and D required the tuberculosis skin test results for the same purpose (Table 2). Only one country required blood tests for the detection of HIV antibodies. Only 10% (1 out of 10 responders) of the new MS reported the requirement of s tuberculosis skin test (TST) for the issuance of seafarer's medical certificate. On the contrary, 28.6% (4 out of 14 responders) from old MS did report that TST is a prerequisite for the issuance of seafarer's medical certificate (p value = 0.18; Table 3). Regarding the validity of the certificates the responders reported a variation from 1 to 5 years. Six out of 21 countries reported that the duration of validity of two years is shortened in elderly seafarers (e.g. >50 years) to only one year.

In addition, only one country reported the requirement of HACCP training for food handlers working on board.

Seven out of 21 of the responders (33%) reported that they identified gaps in occupational health services focused on communicable diseases (Table 1). Responding authorities from Group A and B had the highest percentage of reported gaps (46.2%) followed by group C and D (12.5%) (p = 0.17; Table 2).

Finally, old MS reported a higher frequency regarding gaps in occupational health services in comparison to new MS (45.5% versus 20% respectively; Table 3). The most commonly reported gap by competent authorities was the medical examinations performed in other countries for the issuance of seafarers' medical certificate. The prevalent example given by the competent authorities was related to the tuberculin skin test. Other reported gaps included the absence of a surveillance system related to infectious diseases associated to maritime occupation and the absence of specific legislation related to health and safety of maritime employees.

## Discussion

This study-in the context of the SHIPSAN project-presents for the first time original information about occupational health legislation and practices of competent authorities related to communicable diseases in seafarers, and gaps in occupational health services provided to seafarers focused on communicable diseases, among EU MS. Differences regarding legislation related to occupational health of seafarers, collection and analysis of surveillance data related to communicable diseases, vaccination recommendations, and medical certificates of seafarers were of special interest for this survey in the context of the EU SHIPSAN project.

The analysis of the results revealed differences among countries with a high volume of traffic from passenger ships as compared to those with a lower traffic as well as new and old EU MS.

Our results showed that 19% of the responding countries have reported the existence of additional institutions next to the national public health institutions where communicable diseases of maritime employees are reported. These countries reported a higher frequency of specific national legislation/recommendations or guidelines related to vaccination of seafarers. Specialized institutions for seafarers' occupational health can provide opportunities for national improvements in the field by conducting studies and by acting as a national reference centre for advice [7]. Our results have shown variation between MS with regard to the existence of specific legislation related to seafarer's occupational health This finding indicates that the legislative framework related to seafarer's occupational health is characterized by heterogeneity among MS. Regarding maritime employees vaccination, our data have shown that MS from priority groups A, and B reported a higher prevalence of already existing and specific national legislation/regulation/guidelines related to vaccination of seafarers in comparison to lower priority groups C and D. This difference (by priority group) could be expected; however, the zero percentage reported by priority groups C and D deserves further attention. Seafarers should receive appropriate immunizations before traveling in order to prevent infections. For example, US CDC suggests a routine annual influenza vaccination program for all crew members [12]. The importance of the administration of all routine and travel-indicated vaccines to seafarers cannot be overstated [13]. Specific guidelines for vaccinations may be supportive to this goal and may encourage responsible bodies to clarify issues of financing concerning vaccinations. On the other hand, the adoption (by all EU MS) of common guidelines regarding vaccination of maritime employees is a very complex issue. For example, the guidelines may change depending on time and risk assessment and the regional and global epidemiological data. In addition, the financial cost of the vaccination is another notable aspect which has to be taken into consideration [14]. Descriptive analysis of the data collected has revealed heterogeneity between MS regarding the issuance of health certificates required in order for seafarers to be employed. The heterogeneity observed is related to the medical examinations, laboratory tests required for the issuance of the certificate and the time frame of the duration of the certificate. There is currently no legal or technical framework produced by the European Council for the criteria of the fitness assessment for seafarers. This results in non-conformity for the fitness examination arrangements to ensure that all EU seafarers are assessed against common criteria. Assessment of seafarers work fitness is per se a complex issue [15,16]. However, it should be mentioned that there is such an ongoing initiative undertaken by International Maritime Health Association (IMHA) in collaboration with International Labour Organization and International Transport Workers Federation (ITF). However, the task to produce common and homogeneous criteria for the evaluation of seafarer's medical fitness for work is very difficult. One fundamental question is: do we need separate European criteria, or rather to modify the existing guidelines produced by WHO? The situation will become even more complex given that in this process various factors have to be involved (e.g. ship-owners, trade unions).

As an indication of the non-conformity of the medical examination between MS our study revealed differences in the use of the tuberculosis test. In particular, only a minority of the new MS and of the old MS have reported the requirement of the skin test for tuberculosis for the issuance of seafarer's medical certificates. This point deserves further attention given the increase of tuberculosis incidence in a worldwide scale [17], and the recruitment of seafarers from endemic countries [5].

The prevalent gap reported by the MS was the medical examination performed in other countries. The Italian Ministry of Transports commented that: "*medical examinations made abroad in non-national seafarers embarked on national ships are sometimes not very reliable and an increase of tuberculosis cases and other less severe but not negligible communicable diseases like chickenpox has been observed n recent times (...)*"[18]. Recently, we experienced outbreaks of tuberculosis among seafarers in cargo ships in European countries (Hadjichristodoulou C. Tuberculosis cases among cargo seafarers in Europe. 2009. Ref Type: Personal Communication). However, the discussion of Tuberculin Skin Test (TST) represents another example of a difficult choice. We acknowledge that classifying countries by existing recommendation regarding TST could be problematic given the variety of Tuberculosis-related epidemiological data (e.g. national incidence of TB; migration trends; risk of transmission).

Of particular interest is the fact that only one country required HIV antibodies test result for the evaluation of seafarer's work fitness. It seems that seafarers are infected through heterosexual relationships ashore [19] and even in the multiethnic crews the cases of transmission of HIV between crew members are relatively rare. Furthermore, the examination of HIV status in seafarers is - under a legal point of view-an extremely complex issue.

In addition, another gap identified by our study was the absence of a surveillance system related to infectious diseases of seafarers. This finding - apart from its importance for the occupational health of seafarers-has serious public health implications given the role of maritime employees in the transnational transmission of infectious diseases. Moreover, the above finding is interesting in the context of the ongoing pandemic A/H1N1 2009.

Our study has certain limitations that needed to be taken in to account when interpreting the results. At first, statistical tests were performed on a small number of observations, and it was difficult to reach statistical significance. Additionally, the categorization of countries to priority groups based on criteria such as number of passenger ships, volume of passengers, and number of ports could include limitations given that we are not able to pay attention on other parameters (e.g. way of transmission, density of population, immune-resistance differences between seafarers and passengers).

## Conclusions

The results of the first European study on occupational health legislation and practices related to seafarers focused on communicable diseases have revealed heterogeneity among EU MS.

In conclusion, our work provides evidence that further discussions at international and European levels could be considered, in order to explore the potential for harmonized initiatives regarding occupational health of seafarers.

## Competing interests

The authors declare that they have no competing interests.

## Authors' contributions

GR participated in the design of the study, data analysis and interpretation of results and drafted the manuscript. VAM participated in the design of the study, data analysis and interpretation of results. CS, and TR, CVM, GN, CLRB participated in questionnaire design, interpretation of results and revision of the manuscript. JK supervised the study, participated in the interpretation of results. CH supervised data collection, data analysis, preparation and revision of the manuscript. The members of the Study Group have participated in data collection and providing comments on the final form of the manuscript. All authors read and approved the final manuscript.

## Supplementary Material

Additional file 1 **Questionnaire**. The file contains the questionnaire used in the cross-sectional study. Click here for file
