# Supplementary material for: Occupational health legislation and practices related to seafarers on passenger ships focused on communicable diseases: results from a European cross-sectional study (EU SHIPSAN PROJECT)
Source: J Occup Med Toxicol. 2010 Feb 10;5:1. doi: 10.1186/1745-6673-5-1 (PMC2833162; doi:10.1186/1745-6673-5-1)
Supplement: Additional file 1 — Questionnaire. The file contains the questionnaire used in the cross-sectional study. [file 1745-6673-5-1-S1.PDF]

PROJECT TITLE: Assessing the usefulness of an EU ship sanitation programme and coordinated action for the control of communicable diseases in cruise ships and ferries.

Work Package Description: 6: Collection, Analysis and Presentation of Data
Work Package Leader: University of Thessaly, Greece

GENERAL GUIDELINES FOR THE COMPLETION OF QUESTIONNAIRE D – *"Occupational Health focused on communicable diseases on board cruise ships and ferries amongst the EU countries"*

- **Questionnaire D** is accompanied by **Annex D**.
- Please tick the correct answer either in the questionnaire or in the Annex as requested.
- The annex is to be used to fill in details regarding **occupational health focused on communicable diseases** in your country**.** Please circle every time which question you are answering and describe synoptically in 2-3 lines the legislation or regulation or guideline stating the title, entry into force and amendment dates. Also please give 3-4 keywords or phrases related to the legislation, regulation or guideline. Finally, please send us an **electronic format of the documents referred to the Annex**. If there is not an electronic format available, please provide the original documents.
- The word **"cruise ship"** indicates a vessel (sea/ river/ lake-going) which carries more than twelve passengers who travel for pleasure.
- The word **"ferry"** indicates a vessel (sea/ river/ lake-going) which carries more than twelve passengers and is designed to move people and, often, vehicles on regular itineraries from one place to another.
- The word **"port"** means a seaport or a port on an inland body of water where ships arrive or depart.
- To assist you with the completion of the questionnaire **specific guidelines** for every question have been attached at the end of the questionnaire. Before answering the questions please read the specific guidelines!
- If in your country there are more than one authority issuing the Maritme Health Certificate then 2 questionnaires must be filled in. In this case please inform us and forward the questionnaire to them.

PLEASE SEND YOUR COMPLETED QUESTIONNAIRES TO :
Ass. Prof. Christos Hadjichristodoulou, SHIPSAN Scientific Coordinator **Email:** xhatzi@med.uth.gr

For further assistance please contact:
Ass. Prof. Christos Hadjichristodoulou
SHIPSAN Scientific Coordinator
Address: SHIPSAN Project, University of Thessaly, Lapithon 6, Larissa, 41221, Greece
Tel: 0030 2410 565259 **Fax:** 0030 2410 258197 **Email:** xhatzi@med.uth.gr

QUESTIONNAIRE (D) - OCCUPATIONAL HEALTH FOCUSED ON COMMUNICABLE DISEASES ON BOARD CRUISE SHIPS AND FERRIES AMONGST THE EU COUNTRIES

PURPOSE OF QUESTIONNAIRE: Collection of information on occupational health focused on communicable diseases on board cruise ships and ferries among the EU countries - for assessing the usefulness of an EU strategy.

PLEASE READ THE SPECIFIC GUIDANCE IN ANNEX D BEFORE COMPLETING THIS QUESTIONNAIRE

RESPONDING AUTHORITY	
☐ National central authority	☐ Regional or Local Authority
a. Name of the authority:<br>b. Telephone of the authority:<br>c. Address of the authority:<br>d. E-mail of the authority:<br>e. Contact person:	

D1. Is there any specific national legislation or regulation or guidelines related to **occupational health of seafarers** except for that of EU or of the International Maritime Organisation (IMO) or of the International Labour Organization (ILO)?

☐ **YES** (PLEASE FILL IN THE DETAILS IN ANNEX D) ☐ **NO** (CONTINUE TO QUESTION D2)

D2. Are there additional institutions next to the national public health institutions where communicable diseases of seafarers are reported to?

☐ **YES** (PLEASE SPECIFY BELOW THE AUTHORITY) ☐ **NO** (CONTINUE TO QUESTION D3)

Name of authority: ………..………

Telephone of authority: …………………………………….**Fax of the authority**……………………..……………………………………

Email of authority: ………………………………………………………………………………………...……………………….……..………

Contact person: ……………………………………………….......... Address of the authority: ………………..……………………………

D3. Are the **surveillance of communicable diseases data** related to seafarers **collected and centrally analysed**?

☐ **YES** (PLEASE SPECIFY BELOW THE AUTHORITY) ☐ **NO** (CONTINUE TO QUESTION D4)

Name of authority: ………………………………………………………………………………………………

Telephone of authority: ……………………………**Fax of the authority**……………………………………………

Email of authority: ……………………………………………………………………………………..…………

Contact person: ……………………………………………….......... Address of the authority: ……………………………………………

D4. Is there any specific national legislation or regulations or guidelines related to **vaccination of seafarers**?

☐ **YES** (PLEASE FILL IN THE DETAILS IN ANNEX D) ☐ **NO** (CONTINUE TO QUESTION D5)

D5. Except for the **vaccinations** required by the International Health Regulations (IHR) are there any additional national recommendations or regulations concerning vaccination of seafarers?

☐ **YES** (PLEASE SPECIFY BELOW THE VACCINES REQUIRED) ☐ **NO** (CONTINUE TO QUESTION D6)

VACCINES REQUIRED:

……………………………………………………………………………………………………

……………………………………………………………………………………………………

……………………………………………………………………………………………………

D6. Is it obligatory in your country to issue a **medical certificate** in order for seafarers to travel on any ship (under national or other flag)?

☐ **YES** (PLEASE SPECIFY BELOW THE AUTHORITY ISSUING THE CERTIFICATE) ☐ **NO** (CONTINUE TO QUESTION D10)

Name of the authority: ……………………………………………… Telephone of the authority:…………………………………

Fax of the authority……………………………………………… Email of authority:……………………………………………

Contact person: ……………………………………………………... Address of the authority: ……………………………………

D7. If your answer to the question above is YES, please specify below the medical examinations required.

PLEASE CHECK MEDICAL EXAMINATIONS REQUIRED:

☐ History of communicable diseases plus clinical examinations
☐ Vision function tests
☐ Hearing function tests
☐ Dental check
☐ Blood tests (Please specify………………………………………………………………………………)
☐ X-rays,
☐ Tuberculin tests
☐ Other, please specify: ………………………………………………………………………………

D8. Do the medical certificates have a time of duration?

☐ **YES** (CONTINUE TO QUESTION D9) ☐ **NO** (CONTINUE TO QUESTION D10)

D9. If your answer to the question above is YES, what is the frame time of duration of the certificate?

……………………………………………………………………………………………………

D10. Are there any special requirements for medical examination of food handlers who are employed to work on ships?

☐ **YES** (Please specify the medical examination) ☐ **NO** (CONTINUE TO QUESTION D12)

……………………………………………………………………………………………………

……………………………………………………………………………………………………

……………………………………………………………………………………………………

……………………………………………………………………………………………………

D11. In your opinion are there any **gaps in occupational health services focused in communicable diseases** related to maritime occupation?

☐ **YES** (Please specify the gaps and outline your suggestions) ☐ **NO**

……

……

……

……

……

……

ANNEX D

PLEASE DESCRIBE SYNOPTICALLY IN 2-3 LINES ANY SPECIFIC LEGISLATION AND /OR STATING THE TITLE, DATE, AMENDMENT DATE AND GIVE 3-4 KEYWORDS OR PHRASES. PLEASE PROVIDE THE ELECTRONIC FORMAT OF THE DOCUMENTS. If there is not an electronic format available, please provide the original documents (Please send us the complete legislation document incorporating all the amendments. Otherwise send the original law document attaching all the standing amendments).

QUESTION (PLEASE SELECT WHICH QUESTION YOU ARE ANSWERING)

TITLE / MAIN CONCEPT:

……

……

ENTRY INTO FORCE: ……………………………………MODIFICATION/ AMENDMENT DATE 1: …………………………………………………
MODIFICATION/ AMENDMENT DATE 2: …………………………………………………

IF **GUIDELINES** PLEASE SPECIFY: ☐ MANDATORY ☐ SCIENTIFIC

KEY WORDS OR PHRASES:

……

……

……

Does this legislation/guideline apply to: ☐ Ships under National flag ☐ Ships under Non National flag ☐ Ship with national itinerary ☐ Ships with international itinerary

QUESTION (PLEASE SELECT WHICH QUESTION YOU ARE ANSWERING)

TITLE / MAIN CONCEPT:

……

……

ENTRY INTO FORCE: ……………………………………MODIFICATION/ AMENDMENT DATE 1: …………………………………………………
MODIFICATION/ AMENDMENT DATE 2: …………………………………………………

IF **GUIDELINES** PLEASE SPECIFY: ☐ MANDATORY ☐ SCIENTIFIC

KEY WORDS OR PHRASES:

……

……

Does this legislation/guideline apply to: ☐ Ships under National flag ☐ Ships under Non National flag ☐ Ship with national itinerary ☐ Ships with international itinerary

QUESTION (PLEASE SELECT WHICH QUESTION YOU ARE ANSWERING)

TITLE / MAIN CONCEPT:

……

……

ENTRY INTO FORCE: ……………………………………MODIFICATION/ AMENDMENT DATE 1: …………………………………………………
MODIFICATION/ AMENDMENT DATE 2: …………………………………………………

IF **GUIDELINES** PLEASE SPECIFY: ☐ MANDATORY ☐ SCIENTIFIC

KEY WORDS OR PHRASES:

……

……

Does this legislation/guideline apply to: ☐ Ships under National flag ☐ Ships under Non National flag ☐ Ship with national itinerary ☐ Ships with international itinerary

QUESTION (PLEASE SELECT WHICH QUESTION YOU ARE ANSWERING)

TITLE / MAIN CONCEPT:

……

……

ENTRY INTO FORCE: ....MODIFICATION/ AMENDMENT DATE 1: ....

MODIFICATION/ AMENDMENT DATE 2: ....

IF **GUIDELINES** PLEASE SPECIFY: ☐ MANDATORY ☐ SCIENTIFIC

KEY WORDS OR PHRASES:

……

……

……

Does this legislation/guideline apply to: ☐ Ships under National flag ☐ Ships under Non National flag

☐ Ship with national itinerary ☐ Ships with international itinerary

QUESTION (PLEASE SELECT WHICH QUESTION YOU ARE ANSWERING)

TITLE / MAIN CONCEPT:

……

……

ENTRY INTO FORCE: ....MODIFICATION/ AMENDMENT DATE 1: ....

MODIFICATION/ AMENDMENT DATE 2: ....

IF **GUIDELINES** PLEASE SPECIFY: ☐ MANDATORY ☐ SCIENTIFIC

KEY WORDS OR PHRASES:

……

……

Does this legislation/guideline apply to: ☐ Ships under National flag ☐ Ships under Non National flag

☐ Ship with national itinerary ☐ Ships with international itinerary

QUESTION (PLEASE SELECT WHICH QUESTION YOU ARE ANSWERING)

TITLE / MAIN CONCEPT:

……

……

ENTRY INTO FORCE: ....MODIFICATION/ AMENDMENT DATE 1: ....

MODIFICATION/ AMENDMENT DATE 2: ....

IF **GUIDELINES** PLEASE SPECIFY: ☐ MANDATORY ☐ SCIENTIFIC

KEY WORDS OR PHRASES:

……

……

Does this legislation/guideline apply to: ☐ Ships under National flag ☐ Ships under Non National flag

☐ Ship with national itinerary ☐ Ships with international itinerary

SPECIFIC GUIDELINES FOR

QUESTIONNAIRE D – *"Occupational Health focused on communicable diseases on board cruise ships and ferries amongst the EU countries"*

D1. "Occupational Health" is the promotion and maintenance of the highest degree of physical, mental and social well-being of workers in all occupations by preventing departures from health, controlling risks and the adaptation of work to people, and people to their jobs.

D2. "Communicable disease" – means an illness due to a specific infectious agent or its toxic products that arises through transmission of that agent or its products from an infected person, animal or inanimate reservoir to a susceptible host; either directly or indirectly through an intermediate plant or animal host, vector or the inanimate environment.

D3. Please provide details on the authority responsible for gathering and analysing the surveillance of communicable disease data on seafarers.

D4. In Annex D provide details (e.g. title, entry into force etc.) of any national legislation or regulations specific to vaccination of seafarers.

D5. Please specify whether you carry out vaccinations other the ones obligatory for travel.

D6. Please tick the answer which is applicable to your situation regarding issuing medical certificates in order for seafarers to travel and provide details on the authority issuing the medical certificate.

"**The medical certificate"** is a certificate ensuring that the seafarers are medically fit to perform their duties at sea and are not suffering from any medical condition likely to endanger the health of other persons on board.

D7. Please tick the medical examinations required in order for a medical certificate to be issued to seafarers. **"Medical Examination"** means the preliminary assessment of a person by a duly qualified medical practitioner or by a person recognized by the competent authority as qualified to issue such a certificate, to determine person's health status and potential public health risk to others, and may include the scrutiny of health documents, and a physical examination when justified by the circumstances of the individual case.

D8. Please tick the answer which is applicable to your situation.

D9. Please specify the frame time duration of the health certificates.

D10. Please specify if there are any special requirements for medical examination of food handlers who are employed to work on ships

D11. "Gaps" means any point that should be legislated, but it is not covered by EU legislation or national legislation or when the legislation does not specify its applicability to cruise ships and ferries.
